# Trends in major risk factors and mortality from main non-communicable diseases in Lithuania, 1985–2013

**DOI:** 10.1186/s12889-016-3387-0

**Published:** 2016-08-04

**Authors:** Abdonas Tamosiunas, Jurate Klumbiene, Janina Petkeviciene, Ricardas Radisauskas, Olga Vikhireva, Dalia Luksiene, Dalia Virviciute

**Affiliations:** 1 Institute of Cardiology, Academy of Medicine, Lithuanian University of Health Sciences, Kaunas, Lithuania; 2 Faculty of Public Health, Academy of Medicine, Lithuanian University of Health Sciences, Kaunas, Lithuania; 3 Department of Epidemiology and Public Health, University College London, London, UK

**Keywords:** Mortality, Risk factors, Trends, Non-communicable diseases, Middle-aged population

## Abstract

**Background:**

This study aimed to assess the trends in the prevalence and levels of risk factors and mortality from main non-communicable diseases in the Lithuanian population aged 45–64 years during 1985 to 2013.

**Methods:**

Data from four general population surveys conducted between 1985 and 2008 were used. All these surveys were carried out in Kaunas city and five randomly selected municipalities of Lithuania. Risk factors measured at each survey included regular smoking, overweight, obesity, arterial hypertension, and high levels of blood lipids. In total, data of 10,719 subjects (4,965 men and 5,754 women) aged 45–64 were analysed. Trends in standardized all-cause mortality and mortality from cardiovascular disease (CVD), coronary heart disease (CHD), and malignant neoplasms were estimated for both sexes by joinpoint regression analysis.

**Results:**

In 1985–2013, some favourable trends were observed in the age-standardized mean levels and prevalence of risk factors and mortality from main non-communicable diseases in the Lithuanian middle-aged population. The mean values of blood lipids (with the exception of triglycerides) and the prevalence of dyslipidemias declined. In women, mean levels of systolic blood pressure and body mass index decreased, while in men, the levels of these factors increased. The prevalence of arterial hypertension and obesity increased in men. The proportion of obese women decreased. Smoking prevalence increased in both men and women. From 2007 to 2008, significant downward trends, which were steeper in women than in men, were observed in all-cause, CVD, and CHD mortality.

**Conclusions:**

Despite the favourable changes in some risk factors and mortality rates, the prevalence of risk factors and mortality from main non-communicable diseases in Lithuania are still high. This indicates the importance of the ongoing primary and secondary prevention and optimal treatment of these diseases.

## Background

Cardiovascular disease (CVD) and cancer are major public health problems in Lithuania, as in many other parts of Europe and the world [1–3]. CVD mortality rates have almost halved in most western European countries over the last forty years [4–6]. From 2000 to 2010, global cancer mortality decreased annually by 1.2 % for males and 0.8 % for females in 60 countries [7].

In Lithuania, CVD and cancer mortality rates among men are higher than in Western European countries [8, 9]. In 2012, mortality rates from CVD were 616.0 per 100,000 men in Lithuania, compared to 253.9 per 100,000 men in 27 countries of the European Union (EU). Mortality rates from malignant neoplasms were 276.6 and 216.0 per 100,000 men, respectively [6]. Similarly to men, more than two-fold higher CVD mortality rates were observed among Lithuanian women, as compared to EU women (340.1 and 165.9 per 100,000 women, respectively), whereas female mortality from malignant neoplasms showed no significant difference between the two compared regions (128.2 per 100,000 women in Lithuania and 127.6 per 100,000 women in EU) [6].

Previous epidemiologic studies have shown that the largest proportion of the reduction in mortality from CVD and other chronic non-communicable diseases can be attributed to improvements in lifestyle and other risk factors, and evidence-based pharmacological and interventional therapies [10–12]. Differences in the extent and timing of the decline in the risk factor prevalence and mortality rates indicate the need for analysing time trends in the prevalence of risk factors and the mortality from chronic non-communicable diseases over different time periods and across geographic regions.

In Lithuania, temporal trends in the prevalence of risk factors and mortality from chronic non-communicable diseases have been explored in several epidemiological studies [13–15]. However, these studies were typically based on limited population groups or regions (either urban or rural), and therefore, did not present the national-level data. The nationally representative Lithuanian Health Behaviour Monitoring focused only on the trends in lifestyle risk factors and self-reported weight status [16]. Mortality rates were not linked to the trends in risk factors [3, 17, 18].

The aim of this study was to estimate the trends in the prevalence and levels of risk factors and mortality from main non-communicable diseases in the Lithuanian population aged 45–64 years during 1985–2013.

## Methods

### Study sample

Cross-sectional surveys of the samples representing the Lithuanian population aged 45–64 years were performed in 1986–1987, 1992–1993, 2001–2002, and 2006–2008. All these surveys were carried out in Kaunas, the second largest Lithuanian city (with a population of approximately 300,000 people), and in five municipalities of Lithuania (Joniskis, Kaisiadorys, Kupiskis, Kretinga, and Varena), with populations ranging from 20,000 to 45,000. These municipalities were randomly selected from the northern, southern, eastern, western, and central parts of Lithuania. For each survey, an independent random sample, stratified by gender and age (four age groups: 45–49, 50–54, 55–59, and 60–64 years), was drawn from the Kaunas population register and from the lists of the inhabitants registered at the primary health care centres of five municipalities. In total, data of 10,719 subjects (4,965 men and 5,754 women) aged 45–64 were analysed. The response rate in the surveys varied between 58.1 and 69.6 %.

All four studies were approved by the Lithuanian Bioethics Committee. All respondents provided written informed consent. Data of individual participants were not reported.

### Health examination of random samples

In each survey, blood pressure (BP), weight, height, and blood lipids were measured using the same methodology. The same questionnaires were used in order to obtain required information.

### Measurements

BP was measured twice, using a mercury sphygmomanometer and appropriately sized arm cuffs on the right arm. The initial measurement was performed after five minutes of rest on the right arm. After two minutes, the second measurement was made. The Korotkoff phase 1 (beginning of the sound) and the fifth phase of Korotkoff (disappearance of the sound) were recorded as systolic and diastolic BP. The mean of two readings was used. Hypertension was defined as mean systolic BP of at least 140 mmHg and/or mean diastolic BP of at least 90 mmHg, or BP <140/90 mmHg using antihypertensive medication for the last two weeks before examination. Weight and height were measured with a calibrated medical scale, and without shoes or heavy clothes. Body mass index (BMI) was calculated as the weight in kilograms divided by the height in meters squared (kg/m^2^). Normal weight was defined as BMI <25.0 kg/m^2^, overweight as BMI 25.0–29.99 kg/m^2^, and obesity as BMI ≥30.0 kg/m^2^.

A venous blood specimen was taken to determine lipid levels. Participants were asked to abstain from food intake at least 12 h before the examination. Lipid levels in blood serum were measured by conventional enzymatic method. All laboratory analyses were made in the certified laboratories which used stringent quality control measures. Dyslipidemias were defined using the following criteria: high total cholesterol level - ≥5 mmol/L; high low-density lipoprotein (LDL) cholesterol level - ≥3 mmol/L; low high-density lipoprotein (HDL) cholesterol level - for men <1.0 mmol/L and for women <1.2 mmol/L; elevated triglyceride level - ≥1.7 mmol/L.

The structured questionnaire included questions regarding the respondent’s age, smoking status, antihypertensive treatment, lipid-lowering treatment, and other factors. The question about the use of lipid-lowering medication was asked in all surveys, except the first one. According to smoking status, participants were classified as never smokers, former smokers, and current smokers (smoking at least one cigarette per day).

We calculated the Systematic Coronary Risk Evaluation (SCORE) values using the high-risk version of SCORE, recommended by the European Society of Cardiology [19]. Respondents were categorized into four groups according to SCORE values: 0, 1–4, 5–9, and ≥10 %.

### Mortality data

The Lithuanian Department of Statistics and the Health Information Centre of the Institute of Hygiene were the primary sources of mortality and population data. All deaths for population aged 45–64 years were recorded from 1985 to 2013. Death cases were classified into following groups based on the underlying cause of death and coded by versions 9 and 10 of the International Classification of Diseases (ICD): 1. CVD mortality included codes 390–458 of ICD-9 and I00-I99 codes of ICD-10; 2. Deaths from CHD included codes 410–414 of ICD-9 and I20-I25 of ICD-10; 3. Deaths from malignant neoplasms included codes 140–208 of ICD-9 and C00-C97 of ICD-10; 4. All-cause mortality was defined as 001-E999 codes of ICD-9 and A00-Z99 codes of ICD-10. Mid-year population estimates for the age group 45–64 have been used in rate calculations.

### Statistical analysis

All statistical analyses were performed using the statistical software package IBM SPSS Statistics 20. The data were weighted to match the age distribution of the Lithuanian population aged 45–64 years in 2008. The continuous variables are presented as means and standard deviations (SD), and categorical variables are expressed as proportions. The differences in age-adjusted means of variables between the surveys were assessed using analysis of variance and Bonferroni multiple comparison tests. A χ^2^ test was used for assessing the differences in proportions.

All mortality rates presented were directly standardized by the European standard population. The 95 % confidence intervals (CI) were calculated assuming the Poisson distribution within the age strata. The standardized mortality rate trends by sex were estimated by joinpoint regression analysis [20]. For all mortality rates of interest (all-cause, CVD mortality, CHD mortality, and mortality from malignant neoplasms), four joinpoint regression models were created. The true number of joinpoints (from 0 to 3) was determined in the permutation test. Standardized rates were used as the dependent variable, whereas the year of death was entered in the model as the independent variable. The estimated regression coefficient (beta) multiplied by 100 was reported as the estimated annual percentage change (APC). The 95 % CI of the APC was obtained in the usual manner, from the standard error of the regression coefficient. Differences in rates at the level of *p* < 0.05, using a two-tailed test, were reported as statistically significant.

## Results

Age-standardized mean levels of risk factors, SCORE values, and the prevalence of main risk factors among Lithuanians aged 45–64 years are presented in Table 1 (men) and Table 2 (women). Age-adjusted mean systolic BP significantly increased from 141.8 mmHg in 1986–1987 to 145.5 mmHg in 2006–2008 in men and significantly decreased from 142.3 mmHg to 139.8 mmHg in women during the same period. In men, the prevalence of arterial hypertension showed a significant increase from 62.7 % in the first survey to 74.0 % in the last survey, while no significant trend was found in women.

**Table 1:** Age-standardized means of risk factors, SCORE values, and prevalence of risk factors in men

Mean values and prevalence	1986–1987	1992–1993	1999–2002	2006–2008	P ANOVA
	*n* = 1391	*n* = 953	*n* = 1096	*n* = 1525	
Mean systolic BP, mmHg	141.8 (20.4)	142.5 (22.2)	140.6 (21.7)	145.5*, **, *** (22.2)	<0.001
Mean BMI, kg/m^2^	27.6 (3.92)	27.1 (4.10)	27.7** (4.64)	28.1*, ** (5.13)	<0.001
Mean total cholesterol, mmol/L	6.12 (1.12)	6.03 (1.23)	5.93* (1.32)	5.62*, **, *** (1.11)	<0.001
Mean LDL cholesterol, mmol/L	4.19 (1.13)	4.04* (1.10)	3.77*, ** (1.30)	3.51*, **, *** (1.03)	<0.001
Mean HDL cholesterol, mmol/L	1.29 (0.45)	1.32 (0.44)	1.41*, ** (0.42)	1.37* (0.42)	<0.001
Mean triglycerides, mmol/L	1.43 (0.82)	1.46 (1.04)	1.66*, ** (1.01)	1.81*, **, *** (1.12)	<0.001
Mean SCORE^a^ value, %	4.75 (4.86)	4.75 (4.94)	4.61 (4.89)	5.25*** (5.36)	0.005
					P from chi squared
Arterial hypertension, %	62.7	63.2	61.7	74.0*, **, ***	<0.001
Regular smoking, %	36.5	34.2	39.4	44.6*, **, ***	<0.001
Total cholesterol ≥5.0 mmol/L, %	85.7	78.9*	76.1*	70.2*, **, ***	<0.001
LDL cholesterol ≥3.0 mmol/L, %	86.1	83.9	70.4*, **	68.2*, **	<0.001
HDL cholesterol <1.0 mmol/L, %	27.9	23.6	14.9*, **	15.0*, **	<0.001
Triglycerides ≥1.7 mmol/L, %	24.8	25.5	36.3*, **	44.6*, **, ***	<0.001
Normal weight, BMI <25.0 kg/m^2^, %	25.8	32.6*	28.4	29.0	
Overweight, BMI 25.0–29.9 kg/m^2^, %	49.0	46.8	44.9	40.6*, **	<0.001
Obesity, BMI ≥30.0 kg/m^2^, %	25.2	20.6	26.7**	30.5*, **	
SCORE^a^ (%):					
0 %	7.4	6.7	8.0	8.8	0.001
1–4 %	54.2	53.9	56.0	47.2*, **, ***	
5–9 %	25.3	27.3	23.4	28.7^***^	
≥10 %	13.1	12.2	12.6	15.3	

*Abbreviations: CVD* cardiovascular disease, *BMI* body mass index, *BP* blood pressure, *SD* standard deviation, *HDL* high-density lipoproteins, *LDL* low-density lipoproteins, *SCORE* Systematic Coronary Risk Evaluation

*p < 0.05 compared to 1986–1987, Bonferroni test

**p < 0.05 compared to 1992–1993, Bonferroni test

***p < 0.05 compared to 1999–2002, Bonferroni test

^a^without CVD, other risk factors presented for all participants

**Table 2:** Age-standardized means of risk factors, SCORE values, and prevalence of risk factors in women

Mean levels and prevalence of factors	1986–1987	1992–1993	1999–2002	2006–2008	P ANOVA
	*n* = 1437	*n* = 1055	*n* = 1343	*n* = 1919	
Mean systolic BP, mmHg	142.3 (22.7)	142.5 (25.2)	137.7*, *** (22.6)	139.8*, **, *** (23.0)	<0.001
Mean BMI, kg/m^2^	30.4 (5.24)	29.0*(5.25)	29.3*(5.82)	28.7*, *** (5.91)	<0.001
Mean total cholesterol, mmol/L	6.42 (1.21)	6.21*(1.33)	6.04*, *** (1.38)	5.68*, **, *** (1.16)	<0.001
Mean LDL cholesterol, mmol/L	4.35 (1.10)	4.26 (1.24)	3.82*, *** (1.34)	3.46*, **, *** (1.06)	<0.001
Mean HDL cholesterol, mmol/L	1.45 (0.46)	1.44 (0.43)	1.55*, *** (0.39)	1.54*, ** (0.39)	<0.001
Mean triglycerides, mmol/L	1.34 (0.64)	1.27 (0.70)	1.51*, *** (0.84)	1.59*, **, *** (0.83)	<0.001
Mean SCORE^a^ value, %	1.45 (1.88)	1.34 (1.73)	1.35 (1.79)	1.66*, **, *** (1.91)	<0.001
					P from chi squared
Arterial hypertension, %	59.1	59.5	50.0*, ***	58.9***	<0.001
Regular smoking, %	2.6	3.2	9.0*, ***	15.7*, **, ***	<0.001
Total cholesterol ≥5.0 mmol/L, %	89.8	83.5*	77.0*, ***	72.2*, **, ***	<0.001
LDL cholesterol ≥3.0 mmol/L, %	89.4	86.9	71.5*, ***	65.4*, **, ***	<0.001
HDL cholesterol <1.2 mmol/L, %	30.5	30.7	17.1*, ***	18.2*, ***	<0.001
Triglycerides ≥1.7 mmol/L, %	21.0	19.8	29.0*, ***	37.2*, **, ***	<0.001
Normal weight, BMI <25.0 kg/ m^2^, %	14.4	24.3*	24.2*	29.1*, **, ***	
Overweight, BMI 25.0–29.9 kg/m^2^, %	35.2	38.5	34.7	35.1	<0.001
Obesity, BMI ≥30.0 kg/m^2^, %	50.4	37.2*	41.1*	35.8*, ***	
SCORE^a^ (%):					
0 %	32.0	33.4	34.9	28.6***	0.008
1–4 %	60.6	59.7	58.8	62.7	
5–9 %	6.4	6.3	5.7	8.1	
≥10 %	0.9	0.7	0.6	0.6	

*Abbreviations: CVD*, cardiovascular disease, *BMI* body mass index, *BP* blood pressure, *SD* standard deviation, *HDL* high-density lipoproteins, *LDL* low-density lipoproteins, *SCORE* Systematic Coronary Risk Evaluation

*p < 0.05 compared to 1986–1987, Bonferroni test

**p < 0.05 compared to 1992–1993, Bonferroni test

***p < 0.05 compared to 1999–2002, Bonferroni test

^a^without CVD, other risk factors presented for all participants

A significant increase in mean BMI in men and a significant decrease in mean BMI in women was observed from 1986–1987 to 2006–2008. The proportion of overweight persons significantly decreased in men and remained relatively stable in women, whereas the prevalence of obesity significantly increased in men and decreased in women over the same period.

Between the first and the last survey, the mean levels of total cholesterol and LDL cholesterol significantly decreased in both men and women. The decrease in the mean level of LDL cholesterol was 16.2 % in men and 20.5 % in women. During the 20-year period, an increase of mean HDL cholesterol level by 0.08 mmol/L in men and by 0.09 mmol/L in women was observed. Age-adjusted mean levels of triglycerides significantly increased in both men and women. The prevalence of high levels of total cholesterol and LDL cholesterol and low level of HDL cholesterol significantly decreased in men and women between the first and the last surveys. Conversely, the proportion of men and women with high levels of triglycerides significantly increased.

Between 1986–1987 and 2006–2008, an increase in the prevalence of regular smoking by 1.2 times in men (from 36.5 to 44.6 %) and by 6 times in women (from 2.6 to 15.7 %) was observed.

In men, the mean SCORE value was significantly higher in 2006–2008, as compared to the survey in 1999–2002. Similarly, in women, the highest mean SCORE value was found in the last survey.

Age-standardized mortality rates in 45–64 year-old Lithuanian men and women and mortality trends between 1985 and 2013 are presented in Tables 3 and 4. In women, all four analysed mortality rates were significantly lower in 2013, as compared to 1985. A similar pattern in differences between mortality rates at the beginning and at the end of the study period was observed in men, with the exception of all-cause mortality rate which did not differ significantly between 1985 and 2013. For the whole study period, only a tendency towards decreasing mortality rates (with the exception of all-cause mortality) was observed in men aged 45–64 years, while the decrease in mortality rates from CVD and malignant neoplasms was statistically significant in women .

**Table 3:** Age-standardized mortality rates^a^ and trends in 45–64 year-old Lithuanian men, 1985–2013

Causes of death		Years				Time	period
		1985		2013		1985 -	2013
	Rate	95 % CI	Rate	95 % CI	APC	95 % CI	*p*-value
All causes of death	1714.7	1671.2–1758.2	1669.6	1628.5–1710.8	+0.30	−1.2–+1.8	0.700
CVD	672.8	645.3–700.4	587.1	562.6–611.7	−0.10	−1.5–+1.4	0.900
CHD	471.2	448.2–494.3	352.4	333.4–371.5	−0.60	−1.9–+1.2	0.400
Malignant neoplasms	426.0	404.1–447.9	377.5	357.8–397.8	−0.50	−1.0–+0.0	0.100

*Abbreviations: APC* annual percent change, *CHD* coronary heart disease, *CI* confidence interval, *CVD* cardiovascular disease

^a^per 100,000 population

**Table 4:** Age-standardized mortality rates^a^ and trends in 45–64 year-old Lithuanian women, 1985–2013

Causes of death		Years				Time	period
		1985		2013		1985 -	2013
	Rate	95 % CI	Rate	95 % CI	APC	95 % CI	p-value
All causes of death	677.0	653.4–700.6	551.0	529.5–572.5	−0.50	−1.6–+0.7	0.400
CVD	245.2	231.0–259.3	154.4	143.1–165.7	−1.4	−2.7– −0.1	<0.05
CHD	114.6	105.0-124.3	68.4	60.9–75.9	−1.4	−3.1–+0.3	0.100
Malignant neoplasms	241.3	227.2–255.5	197.8	184.9–210.7	−0.4	−0.8– −0.0	0.041

*Abbreviations: APC* annual percent change, *CHD* coronary heart disease, *CI* confidence interval, *CVD* cardiovascular disease

^a^per 100,000 population

In men, three points of changes in the all-cause mortality trend were detected during the whole study period (Table 5). The periods 1985–1995 and 2000–2007 showed a significant increase in male all-cause mortality, while decreasing mortality rates were found in the period 2008–2013. The similar pattern of all-cause mortality trends for the period 1985–2013 was observed in women. The steepest significant annual increase of all-cause mortality was observed in 1985–1995 (APC = +4.4 % for men and APC = +2.3 % for women), while the steepest significant annual decline of mortality was observed in 2008–2013 for men (APC = −4.9 %) and in 1996-2000 for women (APC = −4.7 %).

**Table 5:** Joinpoint regression analysis of age-standardized mortality rates in 45–64 year-old Lithuanian men and women, 1985–2013

Men					Women				
Joinpoints (Years)	Time period	APC	95 % CI	p-value	Joinpoints (Years)	Time period	APC	95 % CI	p-value
All-cause mortality									
1995	1985–1995	+4.4	+3.0–+5.8	<0.001	1995	1985–1995	+2.3	+1.2–+3.4	<0.001
1999	1996–1999	−6.3	−13.9–+2.0	0.123	2000	1996–2000	−4.7	−8.9– −0.2	0.040
2007	2000–2007	+2.7	+0.4–+5.1	0.022	2007	2001–2007	+2.4	−0.1–+4.9	0.055
	2008–2013	−4.9	−7.6– −2.2	0.001		2008–2013	−4.6	−6.9– −2.3	0.001
CVD mortality									
1994	1985–1994	+4.2	+2.7–+5.8	<0.001	1995	1985–1995	+1.9	+0.6–+3.2	0.005
1998	1995–1998	−7.6	−14.9–+0.2	0.056	2000	1996–2000	−7.6	−12.3– −2.7	0.005
2007	1999–2007	+2.7	+0.9–+4.5	0.006	2006	2001–2006	+3.4	−0.3–+7.2	0.071
	2008–2013	−5.0	−7.6– −2.4	0.001		2007–2013	−5.3	−7.4– −3.3	<0.001
CHD mortality									
1994	1985–1994	+4.3	+2.9–+5.8	<0.001	1995	1985–1995	+3.1	+1.6–+4.7	0.001
1998	1995–1998	−11.1	−17.6– −4.0	0.005	1999	1996–1999	−15.2	−23.1– −6.4	0.002
2006	1999–2006	+2.3	+0.5–+4.0	0.01	2006	2000–2006	+4.4	+1.0–+7.9	0.014
	2007–2013	−4.0	−5.9– −2.0	0.001		2007–2013	−4.9	−7.4– −2.4	0.001
Mortality from malignant neoplasms									
1993	1985–1993	+2.1	+1.3–+2.8	<0.001	1994	1985–1994	+0.5	−0.5–+1.6	0.313
2002	1993–2002	−1.8	−2.5– −1.0	<0.001		1995–2013	−0.9	−1.2– −0.5	<0.001
2007	2003–2007	+0.7	−1.5–+2.9	0.513					
	2008–2013	−2.9	−4.0– −1.8	<0.001					

*Abbreviations: APC* annual percent change, *CI* confidence interval

In men, CVD mortality rates significantly increased in 1985–1994 and 1999–2007, while among women, a significant increase in mortality rates was observed only in 1985–1995. The steepest annual decline in mortality from CVD was found among men in 2008–2013 (APC = −5.0 %) and among women in 1996–2000 (APC = −7.6 %).

CHD mortality rates changed the trend direction from increasing to decreasing twice during the study period in both sexes. In men, the highest annual increase in CHD mortality was observed in 1985–1994 (APC = +4.3 %), while in women, a similar increase in mortality was found in 2000–2006 (APC = +4.4 %). The steepest annual decline in CHD mortality was observed in men during the period 1995–1998 (APC = −11.1 %, *p* = 0.005), and in women, during the period 1996–1999 (APC = −5.2 %, *p* = 0.002).

In men, mortality from malignant neoplasms changed the trend direction twice during the study period, in 1993 and 2007, while in women, only once, in 1994. The steepest annual increase in mortality from malignant neoplasms was observed among men in 1985–1993 (APC = +2.1 %, *p* < 0.001), while the steepest decline of this mortality rate was registered in 2008–2013 (APC = −2.9 %, *p* < 0.001). In women, mortality from malignant neoplasms showed a continuously decreasing trend starting from 1994 (APC = −0.9 %, *p* < 0.001).

## Discussion

This is the first large nationwide study to address the trends in the prevalence of the main risk factors of chronic non-communicable diseases and mortality rates in Lithuania over a period of more than 20 years. We determined gender-specific trends in the mean levels and the prevalence of risk factors in the Lithuanian population aged 45 to 64 years from 1986 to 2008. Both all-cause and cause-specific mortality for Lithuanian men and women were analysed from 1985 to 2013.

In the male population, we observed a significant increase in the prevalence of arterial hypertension and in the mean values of systolic BP. The increasing trends in the mean level of systolic BP contradict the global trends during a similar period. The analysis of data from 199 countries showed that the mean systolic BP decreased by 0.8 mmHg per decade in men and by 1.0 mmHg in women between 1980 and 2008. In Western Europe, systolic BP decreased by 2.0 mmHg per decade in men and by 3.5 mmHg or more in women [21]. The increasing prevalence of obesity and sedentary lifestyle are usually indicated as factors contributing to the adverse trend in arterial hypertension [22, 23]. Our data confirmed the increase in the mean BMI of Lithuanian men. During the period 1983–2008, the prevalence of physical inactivity did not show a statistically significant trend for the Lithuanian urban population aged 45–64 years; however, the proportion of population with low levels of physical activity was high [13]. Other important risk factors for arterial hypertension are alcohol consumption and high sodium intake [24, 25]. The data of the Lithuanian Health Behaviour Monitoring among the population aged 20–64 showed that alcohol consumption had increased over the last two decades, especially among women [16]. High consumption of salt was typical for the majority of the Lithuanian population during the entire study period (daily intake was approximately 4 g in men and 2.5 g in women) [26].

Over the study period, the prevalence of regular smoking increased (by six times in women). Our data are in line with the findings of the Lithuanian Health Behaviour Monitoring that showed the increasing trends in the prevalence of daily smoking up to the year 2000, particularly among women [16]. In Soviet times, smoking prevalence in Lithuania was traditionally low among women, because it was socially unacceptable [13]. After regaining independence in 1990, aggressive marketing of tobacco products contributed to the increasing trends of smoking prevalence mainly among young women [16]. Possibly, those women who started smoking at the age of 20–30 years in the 1990s continued to be smokers at the time of the last survey in 2006–2008, which resulted in the greatest increase in the prevalence of female smoking. According to the Lithuanian Health Behaviour Monitoring data, since 2002, a decreasing trend in smoking has been observed among men, and the increase of smoking prevalence levelled off among women [16]. The decline in the prevalence of regular smoking could be predominantly attributed to tobacco control policies implemented in Lithuania, including advertising bans, bans of smoking in public areas, increased tobacco taxation, and other measures [27, 28].

In our study, we found an increasing trend in the prevalence of obesity and a decreasing trend in the prevalence of overweight in men, whereas in females, a decreasing trend in the prevalence of obesity was observed. Globally, the prevalence of overweight increased from 24.6 % in 1980 to 34.4 % in 2008 [29]. The prevalence of obesity increased by 1.9 times (6.4 % in 1980 vs. 12 % in 2008). In the Soviet era, the prevalence of obesity was particularly high in Lithuania. According to the data from the WHO MONICA study (1983–1985), mean BMI of the Lithuanian population was the highest among 34 populations participating in the study [30]. After regaining independence in the early 1990s, significant economic, social, and psychological problems had an impact on the lifestyle and body weight of Lithuanians. Our data revealed that between 1986 and 1993, the prevalence of obesity decreased from 25.2 to 20.6 % in men and from 50.4 to 37.2 % in women. In subsequent years, the growing economy improved living conditions in the country. The availability and variety of foods, including low-cost energy-dense foods, increased considerably. Dietary surveys showed some positive changes in the nutrition habits of Lithuanians, such as a decrease in the consumption of animal fat and fatty milk products, as well as an increase in the intake of fresh vegetables and fruits [31]. These changes were more common in women than in men, partly explaining sex differences in the time trends of overweight and obesity over the last 15 years. According to the self-reported data of the Lithuanian Health Behaviour Monitoring, the proportion of obese men aged 25–64 increased from 10.7 % in 1994 to 19.5 % in 2014, while in women, the prevalence of obesity was similar in the first and the last surveys, 19.2 and 17.3 %, respectively [32]. Due to changes in the Lithuanian economic sector, demand for physical activity in the workplace decreased, while leisure-time physical activity did not change substantially [13, 16]. Furthermore, the proportion of Lithuanians walking or riding a bicycle to and from work decreased because of the use of private cars [16]. The decrease in physical activity may contribute to the increase in the prevalence of obesity in men.

The favourable changes in mean levels of lipids among both Lithuanian men and women correspond to the global changes in these factors. Globally, mean total cholesterol levels showed a decreasing trend by less than 0.1 mmol/L per decade for both men and women between 1980 and 2008 [33]. The positive changes in lipid levels could be attributable to the changes in dietary intake. Our previous study demonstrated the favourable trends in fatty acid composition of the diet of Lithuanians, caused by increased use of vegetable oil for cooking and replacement of butter spread with margarine [14]. The percentage of energy from saturated fatty acids has decreased from 18.0 to 15.1 % among men and from 17.6 to 14.8 % among women over the period of 20 years. Total dietary effect accounts for a 0.26 mmol/l (43.3 %) decline in serum cholesterol in men and a 0.31 mmol/l (50.8 %) decrease in women [14].

A decreasing trend in all-cause, CVD, and CHD mortality was determined among both Lithuanian men and women, starting from 2007 to 2008. Mortality from malignant neoplasms started to decrease from 1995 in women and from 2007 in men. In Europe, decreasing trends in both all-cause and CVD mortality were observed much earlier than in Lithuania [6, 34, 35]. Most researchers trying to explain the decline in mortality from CVD and CHD suggest that favourable changes of risk factors explain this decline to a greater extent, as compared to treatments of these diseases. For example, approximately 60 % of the CHD mortality decline in the U.K. between 1981 and 2000 was attributable to the reduction in major risk factors and 40 % to combined effects of modern treatments [34]. From years 1969–1972 to 2012, CHD mortality decreased by 82 % in Finnish men and by 84 % in Finnish women aged 35–64 years [36]. During the first 10 years of the study, changes in systolic blood pressure, cholesterol level, and smoking contributed to nearly all of the observed mortality reduction. In the last 10 years of the study, 69 % of the reduction in men and 66 % in women could be explained by changes in those three main risk factors.

In Lithuania, both favourable and unfavourable trends in the prevalence of risk factors were found. More positive changes were observed in the risk profile of women. This fact might partially explain the gender differences in the mortality trends. In Western countries, CVD mortality has declined continuously since the 1960s [37]. On the contrary, the data of joinpoint regression analysis revealed fluctuating mortality trends in the Lithuanian population. The periods of increase and decrease in CVD mortality closely corresponded to the trends in risk factors. Decreasing trends in mortality from malignant neoplasms contradict increasing trends in smoking prevalence. The negative effect of smoking might occur later, particularly in women.

The study period was characterised by dramatic political, economic, and social changes in Lithuania, which increased the gap between socio-economic groups [38, 39] The previous studies carried out in Lithuania demonstrated a strong association between socio-economic determinants and mortality rates [2, 17, 40, 41]. Higher mortality rates in lower socio-economic groups might be caused by such underlying factors as lifestyle, access to health care, and health promotion measures. Socio-economic differences in lifestyle habits (diet, physical activity, smoking, and alcohol consumption) of Lithuanians were revealed in several studies [16, 31, 32]. Thus, a strong link between mortality and alcohol consumption trends has been demonstrated [42, 43]. The decline in mortality observed in Lithuania from 1995 stopped in 2000, when alcohol taxes were decreased resulting in increased alcohol consumption [43]. Alcohol consumption causes considerable social and health problems in the country, including acute effects of alcohol on the risk of CVD death [42].

The strength of our study is that we analysed trends of mean levels and prevalence of risk factors (both lifestyle and biological) and mortality (both all-cause and cause-specific) using nationally representative data for a period of over two decades. Some limitations should be considered when interpreting our findings. First, the present study is limited due to its cross-sectional nature. Consequently, causes and effects could not be established directly. Second, other variables and risk factors, such as socio-economic status, physical activity, and dietary factors, which may have an impact on mortality rates, were not considered. However, our study primarily focused on the trends in more proximal risk factors of chronic non-communicable diseases (such as blood pressure, blood lipids, and smoking), since these risk factors explain, to a large degree, the association between higher mortality and more distal health determinants, such as socio-economic status [44]. Third, the age group included in our study for the analysis of trends in the prevalence and mean levels of risk factors is relatively narrow: 45 to 64 years. The age range in different surveys varied from 25 to 72 years, but only the age group 45–64 years was suitable for the analysis throughout the whole study period. Finally, the response rates that did not exceed 70 % might have resulted in selection bias and potential underestimation of the risk factor levels and prevalence, if non-response was associated with a more adverse risk profile. While the strength of this association is problematic to assess (data on risk factors in non-responders were not available), its magnitude was unlikely to change substantially between the surveys. This, together with the fact that the response rates were relatively similar over time, minimizes the potential impact of non-response on our estimates of the trends in risk factors.

## Conclusions

Some favourable trends in the risk profile and mortality of the Lithuanian population aged 45–64 were determined between 1985 and 2013. Despite these favourable changes, the prevalence of risk factors and mortality from main non-communicable diseases in Lithuania are still high. Therefore, appropriate primary and secondary prevention, as well as optimal treatment of these diseases, remain important health policy goals.

## Abbreviations

APC, annual percentage change; BMI, body mass index; BP, blood pressure; CHD, coronary heart disease; CI, confidence interval; CVD, cardiovascular disease; EU, European Union ; HDL, high-density lipoproteins; ICD, International Classification of Diseases; LDL, low-density lipoproteins; MONICA, Multinational Monitoring of Trends and Determinants in Cardiovascular Disease; SCORE, Systematic Coronary Risk Evaluation; SD, standard deviation; U.K., United Kingdom; WHO, World Health Organization.
